# SafeCodeRL: Security-Constrained Multi-Agent Reinforcement Learning for Trustworthy LLM-Generated IoT/CPS Software

**DOI:** 10.3390/s26113502

**Published:** 2026-06-02

**Authors:** Zhihua Wang, Junfan Chen, Zixiang Wei, Lan Lin, Guoxiang Tong

**Affiliations:** 1 School of Optical-Electrical and Computer Engineering, University of Shanghai for Science and Technology, 516 Jungong Road, Shanghai 200093, China255350806@st.usst.edu.cn (J.C.); 243350753@st.usst.edu.cn (Z.W.); 2 Department of Biomedical Engineering, College of Chemistry and Life Science, Beijing University of Technology, Beijing 100124, China; lanlin@bjut.edu.cn

**Keywords:** large language models, secure code generation, IoT security, cyber-physical systems, trustworthy AI, multi-agent systems, constrained reinforcement learning, sensor networks, edge computing, software vulnerability mitigation

## Abstract

Internet of Things (IoT), sensor-network, and cyber-physical system (CPS) software increasingly relies on large language models (LLMs) and autonomous agents for code generation, maintenance, and vulnerability repair. However, LLM-generated edge services, telemetry APIs, configuration handlers, and data-aggregation routines can introduce SQL injection, path traversal, command injection, hard-coded credentials, and unsafe device-control logic, which may compromise sensing data integrity and system safety. Existing approaches largely rely on static post hoc analysis and lack a unified modeling of the generation process, making it difficult to achieve a principled trade-off between functionality and security. To address this challenge, we propose SafeCodeRL, a framework that integrates multi-agent collaboration with constrained reinforcement learning for trustworthy LLM-generated IoT/CPS software. SafeCodeRL models code generation as a security-aware sequential decision process, where Planner, Code, Security, Test, and Critic agents jointly optimize task decomposition, code synthesis, vulnerability auditing, and sandbox-based validation. We design a constraint-aware policy based on Proximal Policy Optimization, augmented with a Lagrangian mechanism and a shielding strategy to explicitly enforce security constraints. Experiments on real-world engineering and security benchmarks, including SWE-bench, SecurityEval, and CyberSecEval, show that SafeCodeRL reduces high-risk vulnerabilities by over 60% while maintaining high functional correctness. A scenario-level IoT/CPS case study further demonstrates that SafeCodeRL substantially improves secure pass rates for sensor telemetry, edge gateway, configuration-management, and data-aggregation tasks, providing a practical path toward trustworthy AI-assisted software development for sensor-driven systems.

## 1. Introduction

Internet of Things (IoT), sensor networks, edge gateways, and cyber-physical systems (CPSs) increasingly depend on software components that collect telemetry, aggregate sensing streams, expose device-management APIs, and trigger control actions. As large language models (LLMs) and autonomous software agents become practical tools for code generation and maintenance, these sensing systems are likely to include more LLM-generated or LLM-repaired code in data ingestion services, gateway scripts, configuration handlers, and lightweight analytics modules [1]. This trend improves development efficiency, but it also expands the software supply-chain attack surface of sensor-driven systems. A single vulnerable telemetry query, unsafe firmware-path handler, or command-line diagnostic routine can compromise sensing-data integrity, leak device states, or affect the availability of a CPS control loop.

The security risk is not hypothetical. Prior studies show that LLM-based coding assistants can generate code containing common weaknesses such as SQL injection, path traversal, command injection, insecure cryptographic usage, and unsafe resource handling [2,3,4]. These weaknesses are especially problematic in IoT/CPS settings because software modules often bridge physical sensing data, network-facing APIs, embedded devices, and cloud services. For example, an unsafe sensor telemetry API can corrupt a database used for downstream monitoring; a path traversal bug in a firmware or configuration endpoint can expose device files; and a shell-injection bug in an edge-gateway diagnostic script can turn a maintenance interface into a remote execution primitive. Therefore, trustworthy AI for IoT/CPS should not only detect attacks after deployment, but also constrain the code-generation process before vulnerable software reaches the sensing infrastructure.

**Figure 1:**
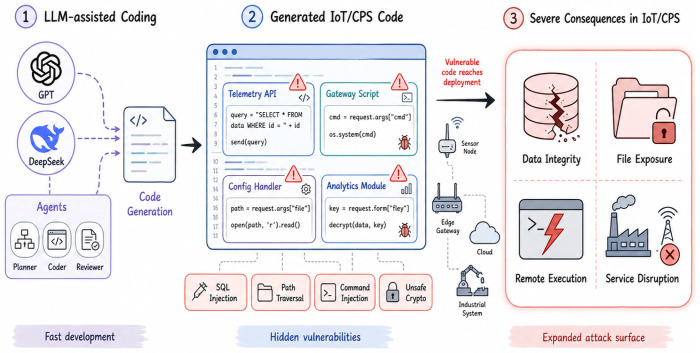
Motivation for security-constrained LLM-generated IoT/CPS software.(The asterisk in the embedded SQL snippet “SELECT * FROM …” is the SQL wildcard for all columns, not a footnote symbol).

Figure 1 visualizes this motivation: LLM/agent-generated IoT/CPS code can transform ordinary software weaknesses into privacy, availability, and physical-safety risks.

Existing secure code generation methods do not fully solve this problem. Post hoc filtering and static-analysis-based rejection can remove some unsafe outputs, but they act after generation and do not shape the underlying policy that repeatedly explores unsafe code patterns [5,6,7]. Fine-tuning and preference-alignment methods internalize secure coding knowledge, but excessive security penalties can cause the alignment-tax problem: the model becomes conservative, incomplete, or less functionally useful [8,9,10]. Multi-agent coding systems improve decomposition and repair by assigning roles to planners, developers, testers, and reviewers [11,12,13], yet most of them still rely on natural-language soft constraints rather than mathematically explicit safety boundaries. For IoT/CPS software, this is insufficient because high-risk actions must be suppressed even when the generator faces ambiguous requirements or adversarial prompts.

To address these limitations, this paper proposes SafeCodeRL, a security-constrained multi-agent reinforcement learning framework for trustworthy LLM-generated IoT/CPS software. SafeCodeRL embeds a collaborative agent workflow into a Constrained Markov Decision Process (CMDP) [14]. The Planner Agent decomposes software requirements and extracts security-relevant context; the Code Agent synthesizes and edits code; the Security Agent reports CWE-level vulnerabilities and severity scores; the Test Agent executes functional and scenario-level tests in a sandbox; and the Critic Agent aggregates heterogeneous feedback into reinforcement learning rewards and routing decisions. By combining Proximal Policy Optimization with Lagrangian safety constraints and action shielding, SafeCodeRL guides the generator toward code that is both functionally correct and security-compliant. The primary methodological novelty of SafeCodeRL is not PPO, CMDP optimization, shielding, or multi-agent collaboration in isolation, but the closed-loop integration of role-specialized agent feedback into a security-aware sequential decision process, where CWE-level audit findings, sandbox test outcomes, and Critic routing decisions are jointly converted into policy-level security costs, Lagrangian penalty adaptation, and action-level shielding within the same generation loop. We therefore position the novelty at the framework-integration level: PPO-style policy updates, Lagrangian CMDP optimization, Shielding, and role-based multi-agent decomposition are adapted from prior safe-RL and the software-agent literature, whereas SafeCodeRL newly couples these elements through CWE-level Security-Agent reports, sandbox Test-Agent traces, and Critic routing so that security evidence directly controls policy costs, adaptive penalties, and action admissibility during LLM code generation.

The main contributions of this work are summarized as follows:

- **We formulate secure LLM code generation as a trustworthy IoT/CPS software problem.** SafeCodeRL targets sensor telemetry APIs, edge-gateway utilities, configuration handlers, and secure data-aggregation routines where generated vulnerabilities can directly affect sensing data integrity and CPS reliability.
- **We propose a multi-agent closed-loop framework that unifies functional testing and security auditing.** The Planner, Code, Security, Test, and Critic agents exchange structured feedback so that generated software is iteratively optimized at both the logical and vulnerability levels.
- **We develop a constraint-aware reinforcement learning algorithm for secure code generation.** The Lagrangian mechanism dynamically balances functionality and security penalties, while action shielding blocks catastrophic code patterns before sandbox execution.
- **We validate SafeCodeRL on established code/security benchmarks and a scenario-level IoT/CPS software suite.** On HumanEval, SWE-bench, SecurityEval, and CyberSecEval, SafeCodeRL improves functional metrics while reducing vulnerability rates; on the 25-task IoT/CPS case study, it improves Secure Pass@1 for telemetry APIs, gateway diagnostics, configuration handling, secure aggregation, and sensor parsing tasks.

The remainder of this paper is organized as follows. In Section 2, we review previous research. Section 3 introduces the SafeCodeRL formulation, agent workflow, and constraint-aware training algorithm. Section 4 details the experimental setup and analyzes benchmark, IoT/CPS, efficiency, ablation, and sensitivity results. Section 5 discusses ethical considerations for secure code generation. Section 6 summarizes limitations and practical implications. Finally, Section 7 concludes the paper and outlines future work.

## 2. Related Work

### 2.1. LLM-Generated Code and Software Vulnerabilities

LLM-based code generation has progressed rapidly from general code models such as Codex, AlphaCode, and Code Llama to agent-based development systems [15,16,17]. However, empirical studies consistently show that generated code may contain exploitable weaknesses. Pearce et al. [2] assessedGitHub Copilot outputs and found frequent security issues.Asare et al. [3] further compared the security of Copilot-generated code with human-written code, while CyberSecEval explicitly evaluates insecure code generation and cyber-risk behaviors of LLMs [4]. These studies motivate treating security not as a post-processing step, but as an optimization target within the generation loop.

### 2.2. IoT/CPS Security and Trustworthy Sensing Software

Security and privacy are long-standing challenges for IoT and sensor-network deployments because heterogeneous devices, networked gateways, and cloud services create multiple trust boundaries [18,19]. Recent generative-AI-enabled IoT studies highlight that LLMs can support device orchestration, application development, and intelligent automation, but they also introduce reliability and security concerns when AI-generated logic interacts with physical sensing infrastructure [1]. Recent work in *Sensors* further illustrates these threads at the sensing-system interface: LLM-assisted modeling supports software security verification in sensor-driven, multi-language settings [20], conversational platforms translate natural language into executable IoT control [21], and MCP-based access to IoT data spaces couples natural-language interaction with server-side authorization to bound LLM-induced misuse [22]. Compared with conventional web or enterprise software, IoT/CPS code often connects data acquisition, local actuation, and remote management. This makes code-level vulnerabilities more consequential because they may affect not only data confidentiality and integrity, but also the availability and safety of sensor-driven services.

### 2.3. Secure Alignment and Reinforcement Learning for Code

Several studies attempt to make LLM-generated code safer through rejection sampling, vulnerability classifiers, supervised fine-tuning, or preference optimization [23,24,25,26]. SVEN, SafeCoder, and CodeUltraFeedback are therefore treated here as model-level secure-alignment references rather than directly comparable workflow baselines: they require method-specific fine-tuning, preference data, or checkpoints and were not designed for the unified inference-time multi-agent protocol or scenario-level IoT/CPS suite used in this paper. Reinforcement learning approaches such as CodeRL and PPOCoder use execution feedback to improve code correctness [27,28]. Nevertheless, most existing methods optimize either functionality or security as a soft preference. SafeCodeRL differs by formulating code generation as a constrained optimization problem, in which security costs are explicitly bounded rather than merely encouraged through prompts or scalar rewards.

### 2.4. Multi-Agent Software Engineering

Multi-agent frameworks such as ChatDev, MetaGPT, AgentCoder, and MapCoder decompose software engineering into collaborative roles and improve iterative refinement [11,12,13,29]. Their main strength is workflow modularity, but their security control is often expressed as natural-language instructions or reviewer feedback. Such soft coordination can be brittle under adversarial prompts, hallucinated critiques, or multi-turn confirmation bias. SafeCodeRL retains the interpretability of multi-agent collaboration while adding CMDP-based safety costs, Lagrangian optimization, and hard action shielding, making the workflow more suitable for high-stakes IoT/CPS software generation.

## 3. Methods

**Figure 2:**
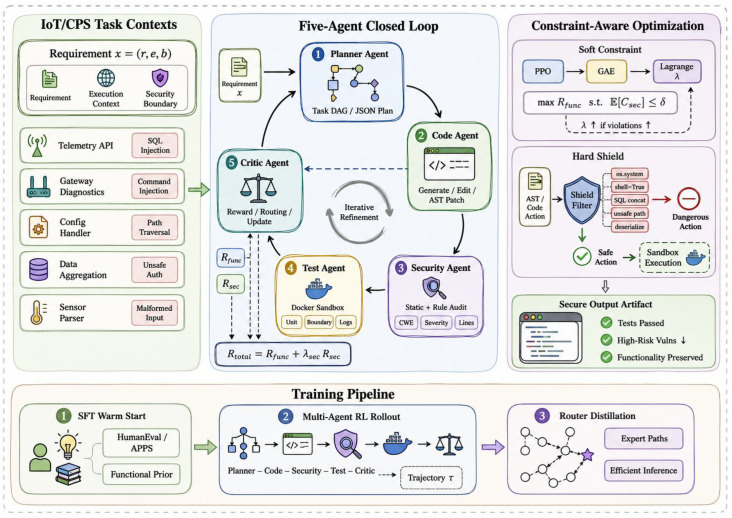
IoT/CPS-oriented overview of the SafeCodeRL framework, including task-context modeling, the five-agent closed loop, constraint-aware optimization, shielding, sandbox execution, and the training pipeline. Solid arrows denote the main generation–feedback flow, dashed arrows denote possible/optional transitions, and the star marks the target (final-goal) node in the Router Distillation module.

This section describes SafeCodeRL as a security-constrained generation framework for IoT/CPS software rather than as a generic coding assistant. As summarized in Figure 2, the framework connects three levels: IoT/CPS task-context modeling, a five-agent closed loop for planning, code generation, security auditing, testing, and critique, and constraint-aware optimization with soft Lagrangian penalties and hard shielding. The following subsections formalize the task representation, agent workflow, reinforcement-learning objective, security constraints, and training pipeline. This design makes security constraints endogenous to the generation policy rather than treating them as post hoc filtering rules or prompt-only soft instructions. In this work, the term IoT/CPS refers to generated software components and deployment-inspired code paths that process sensor telemetry, gateway diagnostics, configuration files, aggregation logic, and networked sensor payloads; it does not imply that the present evaluation constitutes a full physical-device, embedded-firmware, protocol-stack, or hardware-in-the-loop benchmark.

### 3.1. IoT/CPS-Oriented System Formulation

Given a natural-language requirement *x* for a sensing-system software component, SafeCodeRL generates a code artifact *y* together with an iterative feedback trajectory. In the IoT/CPS setting, *x* may describe a sensor telemetry ingestion API, an edge-gateway diagnostic script, a firmware or configuration-file handler, a secure data-aggregation routine, or a networked sensor parser. Table 1 summarizes the five IoT/CPS software contexts considered in this work, together with the typical generated artifacts, the dominant security risks constrained by SafeCodeRL, and the corresponding functional validation signals; this taxonomy is used throughout the paper to anchor the formulation in concrete sensing-system scenarios. These tasks share a common structure: the generated software must satisfy functional tests over representative sensor inputs while avoiding vulnerabilities that may cross the sensing, edge, cloud, or device-control trust boundaries. The corresponding validation therefore focuses on code-level behavior under representative, sandbox-reproducible software inputs rather than on physical sensor fidelity, embedded firmware timing, or live fieldbus/protocol traffic.

We formalize each generation instance as a tuple x=(r,e,b), where *r* is the functional requirement, *e* is the execution context such as data schemas, device interfaces, storage backends, and sandbox limits, and *b* is the security boundary including disallowed APIs, CWE categories, and severity thresholds. The Code Agent produces an action $a_{t}$ corresponding to code generation or code editing. The Test Agent evaluates functional behavior under benchmark tests and mocked sensor streams, while the Security Agent maps the code to a vulnerability set V containing CWE identifiers, affected lines, severity scores, and mitigation hints. The Critic Agent then aggregates these signals into a constrained optimization objective: maximize functional utility subject to an expected security cost bound. This formulation naturally connects IoT/CPS software assurance with CMDP-based policy optimization.

### 3.2. Five-Agent Architecture

One of the key strengths of SafeCodeRL lies in its collaborative multi-agent workflow, where specialized roles are assigned to distinct tasks. The five agents form a closed-loop pipeline of generation, evaluation, feedback, and optimization. First, the Planner agent interprets user requirements expressed in natural language and decomposes them into sub-tasks while preserving IoT/CPS context such as sensor schemas, edge APIs, and security boundaries. These sub-tasks are handled by the Code agent to produce an initial code draft. Next, the generated code sequentially passes through the Security and Test agents for vulnerability assessment and functional verification. Finally, the Critic agent aggregates all dynamic feedback, computes reinforcement learning rewards, and guides the Code agent to perform targeted code revisions or directly updates the global policy network. The specific design of each agent is described below.

#### 3.2.1. Planner Agent

The Planner agent serves as the cognitive vanguard of the entire architecture. Directly generating complete code from complex user prompts often leads to logical inconsistencies. To address this, the Planner employs a few-shot chain-of-thought prompting strategy [30], mapping high-level development requirements T into a sequence of fine-grained, executable sub-tasks. For IoT/CPS tasks, the Planner also extracts sensor-payload assumptions, device or gateway interfaces, storage resources, and explicit security constraints. These sub-tasks are output in a standardized JSON structure, for example

[{"task_id": i, "desc": "...", "in_out_types": "..."}].

Internally, the Planner maintains a directed acyclic graph representing task dependencies and performs topological sorting to ensure that sub-tasks are executed in strict accordance with the control flow of the code. This design significantly reduces the complexity of downstream code generation.

#### 3.2.2. Code Agent

The Code agent functions as the core generation engine, responsible for implementing the sub-tasks defined by the Planner. Code generation follows a dynamic temperature decay strategy, initially producing function stubs and implementations according to the Planner’s JSON specification. A key feature of the Code agent is its self-refinement mechanism [31], which enables iterative improvement of generated code. When feedback triggers a rewrite, the agent applies abstract syntax tree-based partial editing, modifying only the affected code segments while preserving previously validated functionality, thus continuously improving code quality without introducing regressions.

#### 3.2.3. Security Agent

The Security agent acts as the system’s critical safety gateway, designed to detect deep-seated vulnerabilities that automatic generation may overlook. Its internal architecture comprises a dual-auditing pipeline. The first stage combines Python 3.11 AST inspection with CodeQL data-flow analysis using the Python security-and-quality query suite and IoT/CPS-oriented custom checks [32] to extract control flow and data flow graphs, identifying potential taint paths [33]. The second stage employs a rule-based heuristic audit to evaluate these paths and filter out false positives from the static analysis by checking source-to-sink reachability, sanitizer use, CWE category, severity level, and affected lines before a finding is passed to the Critic Agent. In IoT/CPS scenarios, this audit emphasizes unsafe telemetry queries, path construction for firmware or configuration files, command execution in gateway diagnostics, insecure authentication for aggregation endpoints, and unsafe parsing of networked sensor messages. The active rule set targets CWE-89 SQL injection, CWE-22 path traversal, CWE-78 command injection, unsafe deserialization, hard-coded credentials, and insecure authentication in telemetry, gateway, configuration-management, aggregation, and parser code. For vulnerability-rate computation, a finding is counted only when it is retained after source-to-sink reachability, sanitizer, CWE-category, severity, and affected-line checks; warnings that are sanitized, unreachable, or not reproducible under the task boundary are treated as false positives and excluded from the reported medium/high-risk vulnerability rate. The final output is a structured set of tuples, including vulnerability identifiers, affected lines, severity scores, and suggested mitigations, which provides precise penalty signals for the Critic agent.

#### 3.2.4. Test Agent

The Test agent is responsible for dynamic validation of the generated code. To ensure safe execution, all tests run in a resource-constrained Docker sandbox [34] with enforced timeouts, memory limits, and prohibition of system-level I/O. The agent executes both user-provided benchmark tests and automatically generated boundary test cases for open-ended tasks. For sensing software, the Test Agent additionally uses mocked sensor streams, malformed telemetry payloads, restricted virtual device trees, and sandboxed gateway commands to approximate deployment-relevant behavior without contacting real devices. For IoT/CPS tasks, the sandbox tests include vulnerability-triggering boundary inputs, such as tainted telemetry strings, path-traversal payloads, command-like gateway parameters, malformed sensor packets, and authentication edge cases, so that static findings can be cross-checked against executable task behavior when a dynamic trigger is available. Its feedback mechanism captures more than simple pass rates; it records complete execution logs, including standard output, standard error, and stack traces, providing detailed context for error analysis and allowing the Code agent to perform targeted refinements.

#### 3.2.5. Critic Agent

The Critic agent serves as the optimization hub of the system, enabling the solution of constrained Markov decision processes through reinforcement learning. Implemented as a parameterized value network, it encodes all state information—including security penalty tuples, execution logs, and the current code AST—into hidden feature vectors. A multi-objective reward function aggregates these features into a comprehensive reward signal, and generalized advantage estimation [35] computes the advantage function to guide policy gradient updates for the Code agent. During inference, a lightweight routing multi-layer perceptron at the top of the Critic outputs a discrete probability distribution over possible actions, including code modification, security retesting, functional retesting, or task termination, thereby managing dynamic scheduling and closed-loop optimization within the system.

The five-agent architecture leverages highly engineered interface constraints and pipeline design to enable each agent to perform its specific role, effectively decoupling tasks that are difficult for a monolithic LLM to handle simultaneously. The Planner agent decomposes complex development requirements into fine-grained sub-tasks, the Code agent performs targeted local code refinements, the Security agent conducts precise taint tracking and vulnerability detection, and the Test agent executes dynamic sandbox testing. The Critic agent consolidates dispersed error logs and security alerts, computing global reward signals through its value network and routing multi-layer perceptron. This architecture enhances the interpretability and controllability of the automated code generation workflow while ensuring that the final output aligns with both functional robustness and security compliance.

### 3.3. Reinforcement Learning Modeling

In reinforcement learning for code generation, directly employing binary feedback, which merely distinguishes between all tests passing or failing, leads to severe sparse reward problems, making it difficult for the policy network to converge during early exploration. To address this issue and explicitly incorporate safety constraints, SafeCodeRL introduces a dense composite reward function:

(1) $$R_{\mathrm{total}}\left(s_{t},a_{t}\right)=R_{\mathrm{func}}\left(s_{t},a_{t}\right)+\lambda_{\sec}\cdot R_{\sec}\left(s_{t},a_{t}\right).$$

The functional reward $R_{\mathrm{func}}$ is designed as a progressive, milestone-based structure, decomposing execution feedback into multiple stages and accumulating rewards via indicator functions I. This encourages the model to gradually master fundamental syntax, runtime robustness, partial correctness, and ultimately full functional compliance:

(2) $$R_{\mathrm{func}}=w_{1}I_{\mathrm{comp}}+w_{2}I_{\mathrm{exec}}+w_{3}\left(\frac{N_{\mathrm{pass}}}{N_{\mathrm{total}}}\right)+w_{4}I_{\mathrm{pass}@k},$$

where $w_{1}=0.2$ corresponds to AST parsing and successful compilation, ensuring that generated code is free from basic syntax errors such as missing indentation or unmatched brackets; $w_{2}=0.3$ reflects crash-free execution, indicating that the code runs without runtime exceptions such as IndexError or TypeError, even if assertions are not yet satisfied; $w_{3}=0.5$ provides continuous reward proportional to the fraction of test cases passed; and $w_{4}=1.0$ represents the ultimate reward when all functional requirements are met (Pass@k). These weights are assigned as a curriculum-style progression rather than independent tuned constants: the reward mass increases from syntactic validity to executable behavior, partial unit-test success, and finally full task completion, so that early learning receives dense guidance without making incomplete programs competitive with fully correct ones.

The security-aware penalty $R_{\sec}$ evaluates not only the presence of vulnerabilities but also their severity. Denoting the set of vulnerabilities reported by the Security Agent as V, the security reward is computed as

(3) $$R_{\sec}=1.0-\min\left(3.0,\sum_{v\in V}\mathrm{penalty}\left(v\right)\right).$$

A baseline positive reward of +1.0 is granted if no vulnerabilities are detected (V=∅). Low-risk issues, such as hard-coded non-sensitive credentials or suboptimal cryptographic configurations, incur a safety cost of 0.2, medium-risk vulnerabilities, including unrestricted filesystem access or potential denial-of-service hazards, incur a cost of 1.0, and high-risk vulnerabilities, which may lead to severe consequences such as SQL injection, remote code execution, or buffer overflows, incur a cost of 2.0; these positive costs are subtracted from the clean-code reward in the security-reward equation above. The cumulative cost is clipped at 3.0 to prevent batches containing multiple findings from producing excessively large negative updates that destabilize PPO optimization.

The weighting factor $\lambda_{\sec}$ is dynamically adjusted as a Lagrange multiplier within the constrained Markov decision process framework. During training, if the model exhibits a high rate of security violations within a batch, $\lambda_{\sec}$ increases automatically, amplifying the contribution of $R_{\sec}$ and enforcing policy updates that prioritize adherence to safety constraints. This dense composite reward design enables the reinforcement learning agent to balance functional correctness and security compliance in a finely graded and explicitly guided manner, mitigating the limitations of sparse binary feedback while promoting safer and more robust code generation. The overall training procedure that integrates the dense functional reward $R_{\mathrm{func}}$, the security-aware penalty $R_{\sec}$, and the dynamically adjusted multiplier $\lambda_{\sec}$ is summarized in Algorithm 1.
**Algorithm 1:** SafeCodeRL: Reinforcement Learning with Functional and Security-Aware Rewards
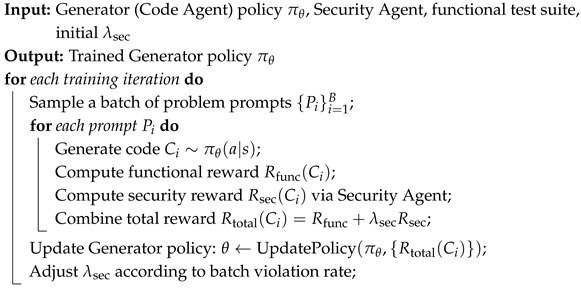


### 3.4. Security Constraint Modeling

To rigorously solve the constrained Markov decision process (CMDP) [14] and balance exploration with safety, SafeCodeRL introduces a dual-constrained optimization strategy combining soft and hard constraints. The soft constraint is based on Lagrangian relaxation [36], addressing the core difficulty of CMDP where policy updates must ensure that the cumulative cost remains within a predefined safety threshold δ. The constrained optimization problem is transformed into an unconstrained min-max dual problem by introducing a penalty term. The Lagrangian function is defined as

(4) $$L\left(\pi_{\theta },\lambda \right)=E_{\tau ∼\pi_{\theta }}\left[\sum_{t=0}^{T}\gamma^{t}R_{\mathrm{func}}\left(s_{t},a_{t}\right)\right]-\lambda \left(E_{\tau ∼\pi_{\theta }}\left[\sum_{t=0}^{T}\gamma^{t}C_{\sec}\left(s_{t},a_{t}\right)\right]-\delta \right),$$

where $R_{\mathrm{func}}$ denotes the functional reward and $C_{\sec}$ the safety cost. Within the PPO actor-critic framework, the policy parameters θ and the Lagrange multiplier λ are updated alternately at each iteration. The multiplier λ is dynamically adjusted using dual gradient ascent:

(5) $$\lambda \leftarrow \max\left(0,\lambda +\alpha_{\lambda }\left({\hat{J}}_{C}\left(\pi_{\theta }\right)-\delta \right)\right),$$

where $\alpha_{\lambda }$ is the learning rate of the multiplier and ${\hat{J}}_{C}\left(\pi_{\theta }\right)$ represents the batch-estimated expected cumulative safety cost. If the generated code frequently violates safety constraints exceeding δ, λ rapidly increases, amplifying the penalty term and forcing the Code Agent to adopt a conservative safe strategy. Conversely, when code generation is sufficiently secure, λ decreases, encouraging exploration of more complex functional implementations. In our repeated runs, the projected dual update kept the multiplier stable after the early exploration phase: across five independent seeds, the final λ converged to 0.18±0.03. Initializing $\lambda_{0}$ in {0.05,0.10,0.20} changed the early penalty strength but produced only small variations in SecurityEval VR (4.4–4.9%) and SWE-bench resolution (13.8–14.2%), indicating that the constraint mechanism is not highly sensitive to the initial multiplier.

While Lagrangian relaxation enforces a soft constraint at the expected-probability level, it may still drive the model to produce catastrophic actions during early training when λ is small. To provide system-level, physically absolute safety guarantees, SafeCodeRL incorporates a hard shielding mechanism [37]. The action space A of the Code Agent is partitioned into a safe subset $A_{\mathrm{safe}}$ and a dangerous subset $A_{\mathrm{danger}}$. The Shield module *S* acts as a filter for the policy, formalized as

(6) $$\pi_{\mathrm{shield}}\left(a_{t}|s_{t}\right)=\left\{\begin{array}{cc} \frac{\pi_{\theta }\left(a_{t}|s_{t}\right)}{\sum_{a\in A_{\mathrm{safe}}}\pi_{\theta }\left(a|s_{t}\right)}, & \mathrm{if}\;a_{t}\notin A_{\mathrm{danger}}\\ 0, & \mathrm{if}\;a_{t}\in A_{\mathrm{danger}}. \end{array}\right.$$

In practice, Shield extracts the abstract syntax tree (AST) of each generated code block and compares it against a known catastrophic blacklist, including patterns such as subprocess.Popen(..., shell=True), SQL string concatenation keywords, os.system, unrestricted path joins, unsafe deserialization calls, and device-control APIs not permitted by the current IoT/CPS boundary *b*. If an action $a_{t}\in A_{\mathrm{danger}}$ is detected, Shield intercepts it before sandbox execution, injects the maximal negative reward into the environment, and enforces resampling through truncated rejection sampling. This mechanism ensures, at the execution level, that high-risk vulnerabilities are not activated in telemetry, gateway, configuration-management, or aggregation tasks, providing a safe operational envelope for code generation. Thus, the robustness mechanism is code- and behavior-facing rather than prompt-facing: obfuscated wording can still lead to interception if the generated artifact reaches monitored sinks or violates the active IoT/CPS boundary.

### 3.5. Synergy and Training Pipeline

To ensure that the model acquires strict safety alignment without degrading its fundamental coding capability, SafeCodeRL employs a rigorous three-stage collaborative training pipeline. In the first stage, supervised fine-tuning (SFT) on high-quality code corpora establishes a robust functional prior for the Code Agent, preventing policy collapse during early reinforcement learning. The Code Agent’s underlying large language model is fine-tuned on the filtered HumanEval [15] and APPS [38] datasets $D_{SFT}$ using the standard causal language modeling objective:

(7) $$L_{SFT}\left(\theta \right)=-E_{\left(x,y\right)∼D_{SFT}}\left[\sum_{i=1}^{\left|y\right|}\log\pi_{\theta }\left(y_{i}|x,y_{<i}\right)\right].$$

This stage ensures that the Code Agent deeply understands the sub-task format provided by the Planner agent and possesses the foundational logic to pass unit tests.

In the subsequent stage, the five-agent system is deployed into a simulated closed-loop environment for multi-agent rollouts. The Code Agent samples code actions $a_{t}$ according to the current state $s_{t}$, sequentially passing through the Security and Test agents to obtain feedback, while the Critic agent collects the full interaction trajectory $\tau =\{\left(s_{t},a_{t},r_{t},s_{t+1}\right)\}$. When the task is IoT/CPS-oriented, the rollout state includes the generated code AST, vulnerability tuples, sandbox traces, sensor-input coverage, and the active boundary specification *b*. Using the generalized advantage estimate (GAE) [35] ${\hat{A}}_{t}$ computed by the Critic, the Code Agent’s policy weights θ are updated through the clipped Proximal Policy Optimization (PPO) [39] objective to achieve safe alignment:

(8) $$L_{PPO}\left(\theta \right)={\hat{E}}_{t}\left[\min\left(\rho_{t}\left(\theta \right){\hat{A}}_{t},\;\mathrm{clip}\left(\rho_{t}\left(\theta \right),1-\epsilon ,1+\epsilon \right){\hat{A}}_{t}\right)\right],$$

where the probability ratio $\rho_{t}\left(\theta \right)=\frac{\pi_{\theta }\left(a_{t}|s_{t}\right)}{\pi_{\theta_{\mathrm{old}}}\left(a_{t}|s_{t}\right)}$, and the hyperparameter ϵ controls the update magnitude to ensure smooth convergence within the trust region during exploration.

Finally, to enhance inference efficiency and prevent overcorrection, the routing multi-layer perceptron (Routing MLP) atop the Critic is trained to dynamically schedule agent calls. Successful trajectories with the highest cumulative rewards are extracted to form an expert path dataset. Let $z_{t}\in \left\{\mathrm{Call}\_\mathrm{Code},\mathrm{Call}\_\mathrm{Sec},\mathrm{Call}\_\mathrm{Test},\mathrm{End}\right\}$ denote the next agent to invoke. The routing network is optimized using an imitation learning cross-entropy loss:

(9) $$L_{Router}\left(\phi \right)=-{\hat{E}}_{\left(s_{t},z_{t}^{*}\right)∼D_{expert}}\left[\sum_{k}z_{t,k}^{*}\log p_{\phi }\left(z_{t,k}|s_{t}\right)\right].$$

With this aligned Critic, the system can autonomously predict the optimal flow based on AST and safety feature vectors after initial code generation, such as bypassing the Security agent if the code is absolutely safe or returning to the Code Agent in case of severe violations, thereby significantly reducing the number of iteration cycles while maintaining both functional correctness and strict safety compliance.

## 4. Experiments

To rigorously and comprehensively assess the effectiveness of SafeCodeRL, we construct an experimental framework that includes general code-generation benchmarks, security benchmarks, efficiency analysis, ablation studies, sensitivity analysis, and an IoT/CPS software security case study. This evaluation matrix is designed to capture both functional correctness and security alignment, while also testing whether the framework can be instantiated in sensor-driven edge software scenarios.

### 4.1. Datasets, Evaluation Metrics, and Base Models

To systematically evaluate the functional and security performance of SafeCodeRL, we employ diverse datasets, clearly defined performance metrics, and analyze the impact of different large language models as base agents. The functional evaluation datasets include HumanEval [15], consisting of 164 fundamental Python programming tasks, and SWE-bench [40], comprising 2294 complex tasks derived from real GitHub engineering issues. The security evaluation datasets include SecurityEval, which contains prompts designed to induce common CWE vulnerabilities [41], as well as CyberSecEval, which assesses cyber-risk behavior and insecure code generation in LLMs [4]. To connect these general benchmarks with the Sensors scope, we further construct a scenario-level IoT/CPS software suite containing 25 tasks across five categories: sensor telemetry database/API logic, edge-gateway diagnostics, firmware/configuration file handling, secure sensor-data aggregation, and networked sensor parsing.

For base models, to investigate the effect of model scale and architecture on SafeCodeRL, we utilize three models as generators and critics: the open-source lightweight model Llama-3-8B [42], the open-source flagship model DeepSeek-V3 [43], and the closed-source state-of-the-art model GPT-4o [44]. Additionally, the large-scale inference model DeepSeek-R1 [45] serves as a baseline for single-agent test-time computation to compare the performance gains of the multi-agent system in terms of both functionality and security. To separate the effect of explicit security constraints from generic multi-agent collaboration, we further evaluate four representative multi-agent code-generation baselines—ChatDev [11], MetaGPT [12], MapCoder [29], and AgentCoder [13]—using GPT-4o as the shared backbone on HumanEval, SWE-bench, SecurityEval, and CyberSecEval.

The evaluation metrics encompass both functional correctness and security compliance. To ensure that our evaluation protocol is consistent with widely recognized practice in the LLM code-generation community rather than self-defined, we directly reuse or minimally extend the metrics established by prior authoritative benchmarks. The Pass@1 (%) metric is adopted as the gold standard for code functional correctness, following the definition introduced together with HumanEval [15], which has since been used as the de facto functional metric in subsequent code LLM studies [16,17]. It is defined as

(10) $$\mathrm{Pass}@1=\frac{N_{\mathrm{correct}}}{N_{\mathrm{total}}}\times 100\%,$$

where $N_{\mathrm{correct}}$ is the number of code submissions that pass all test cases and $N_{\mathrm{total}}$ is the total number of evaluated tasks. Vulnerability Rate (VR, %) quantifies the proportion of generated code containing high- or medium-severity security vulnerabilities, and is adopted in line with the evaluation conventions of SecurityEval [41] and CyberSecEval [4], both of which report the share of generated programs that contain insecure CWE patterns. Formally,

(11) $$\mathrm{VR}=\frac{N_{\mathrm{vulnerable}}}{N_{\mathrm{total}}}\times 100\%,$$

where $N_{\mathrm{vulnerable}}$ denotes the number of code samples with at least one detected vulnerability. Resolution Rate (%) measures the proportion of successfully resolved SWE-bench issues, exactly following the official SWE-bench evaluation protocol [40]:

(12) $$\mathrm{Resolution}\;\mathrm{Rate}=\frac{N_{\mathrm{resolved}}}{N_{\mathrm{total}}}\times 100\%,$$

where $N_{\mathrm{resolved}}$ indicates the number of issues fully fixed by the model. For the IoT/CPS case study, we additionally report Secure Pass@1, which is a security-aware extension of the standard Pass@1 metric [15] inspired by the joint functional-and-security evaluation perspective advocated in CyberSecEval [4]. It is defined as the proportion of tasks that both pass functional tests and contain no medium- or high-severity vulnerability:

(13) $$\mathrm{Sec}\mathrm{ure}\;\mathrm{Pass}@1=\frac{N_{\mathrm{correct}\cap \sec\mathrm{ure}}}{N_{\mathrm{total}}}\times 100\%.$$

All stochastic generation experiments were repeated five times with independent random seeds. Table 2 and Table 3 report the resulting mean point estimates, while selected confidence intervals and paired task-level permutation tests are reported in the text for the most relevant comparisons. This reporting is particularly important for the compact 25-task IoT/CPS suite, where small percentage differences should not be over-interpreted from a single run.

These four metrics collectively form a multi-dimensional evaluation framework that is grounded in established benchmarks [4,15,40,41] rather than constructed ad hoc, allowing SafeCodeRL’s performance to be assessed not only in terms of functional correctness but also in terms of safety compliance and its capability to handle complex engineering and sensing-system tasks.

### 4.2. Implementation Details

To ensure the rigor and full reproducibility of our experiments, we provide a detailed description of the system configuration and hyperparameter settings for SafeCodeRL. All model fine-tuning and multi-agent rollouts were conducted on compute nodes equipped with eight NVIDIA A100 GPUs, each with 80 GB memory. To maximize inference throughput, open-source base models were deployed with tensor parallelism using the vLLM framework [46]. The multi-agent collaborative pipeline was built on top of the AutoGen framework [47], with extensive customizations to support SafeCodeRL’s specialized five-agent interactions.

For code generation, the Code Agent employs an initial decoding temperature of T=0.8, with a dynamic linear decay strategy applied across successive interaction iterations, decreasing by 0.15 per round down to a minimum of 0.2. This balances early-stage exploration diversity with later-stage strict determinism required for vulnerability mitigation. The maximum generation length (Max New Tokens) is set to 2048, and the maximum number of allowed interaction iterations is capped at k=4.

Reinforcement learning hyperparameters for PPO and CMDP optimization were configured as follows: the policy network (Actor) learning rate is set to $1\times 10^{-5}$, while the value network (Critic) learning rate is $5\times 10^{-5}$, both employing a cosine annealing scheduler with linear warmup. The PPO clipping parameter is ϵ=0.2, the discount factor is γ=0.99, and the generalized advantage estimation parameter is $\lambda_{\mathrm{GAE}}=0.95$. For the Lagrangian soft-constraint mechanism, the initial multiplier is set to $\lambda_{0}=0.1$, with a gradient step size of $\alpha_{\lambda }=0.01$, and a tolerated expected safety cost threshold of δ=0.15. The threshold δ=0.15 allows a small batch-level residual safety cost during exploration while still forcing $\lambda_{\sec}$ to increase once violations become systematic, matching the security-first objective of the benchmark setting.

The Test Agent executes within a highly restricted Docker sandbox environment [34], designed to enforce rigorous security isolation. To prevent system escapes during code execution, the sandbox imposes a 10 s hard timeout, a 512 MB memory limit, and disables all external network requests and unauthorized low-level system calls. For the IoT/CPS case study, we do not retrain any base model; instead, we instantiate the same inference-time SafeCodeRL workflow using mocked sensor payloads, synthetic gateway requests, virtual configuration paths, and boundary tests for malformed network messages. This configuration ensures that dynamic testing is conducted safely while faithfully capturing functional and security-relevant feedback for reinforcement learning updates. Mocked payloads and virtual paths are used deliberately to make the evaluation reproducible and safe for generated code that may contain injection, traversal, or unsafe command-execution behavior; they should be interpreted as code-level validation inputs rather than substitutes for real sensors, embedded firmware, or live MQTT/CoAP traffic.

### 4.3. Main Results

#### Evaluation of Base Models Under SafeCodeRL

We deployed various base models within the SafeCodeRL framework and conducted a comprehensive five-run comparison against single-agent zero-shot generation, Self-Refine [31], a reasoning-model baseline, and four generic multi-agent code-generation baselines, as summarized in Table 2. This benchmark-level comparison directly separates generic multi-agent orchestration from SafeCodeRL’s explicit security-constrained optimization while reducing dependence on a single stochastic run.

The results highlight several key advantages of the SafeCodeRL framework. First, generic multi-agent baselines improve functional performance over single-agent and self-refinement baselines: with GPT-4o as the backbone, ChatDev, MetaGPT, MapCoder, and AgentCoder reach 88.6–90.2% HumanEval Pass@1 and 11.6–13.1% SWE-bench resolution. However, their residual vulnerability rates remain high, ranging from 17.6% to 21.9% on SecurityEval and from 16.4% to 19.8% on CyberSecEval. This indicates that multi-agent collaboration alone improves problem decomposition and iterative repair, but does not provide sufficiently strong security control.

Second, SafeCodeRL consistently outperforms the strongest generic multi-agent baseline. Compared with AgentCoder, SafeCodeRL with GPT-4o improves HumanEval Pass@1 from 90.2% to 91.4% and SWE-bench resolution from 13.1% to 14.8%, while reducing SecurityEval VR from 17.6% to 5.5% and CyberSecEval VR from 16.4% to 6.2%. SafeCodeRL with DeepSeek-V3 further achieves the lowest SecurityEval VR of 4.2% and CyberSecEval VR of 5.8%. These results indicate that SafeCodeRL’s security gains are not merely a consequence of using multiple agents, but arise from coupling role-specialized collaboration with CWE-level auditing, CMDP-based security costs, Lagrangian optimization, and hard action shielding.

Finally, the empirical improvements emphasize the value of integrating multi-round agent interaction with explicit security constraints. The staged functional rewards encourage progressive code repair, whereas the Security Agent, Lagrangian penalty, and Shielding module suppress unsafe actions that generic multi-agent workflows may still allow. Therefore, the comparison supports a more precise conclusion: SafeCodeRL benefits from multi-agent collaboration, but its main advantage over existing multi-agent coding systems comes from security-constrained optimization rather than workflow modularity alone.

Overall, these results validate the efficacy of the SafeCodeRL framework in enhancing both functional robustness and security alignment across a spectrum of base language models, underscoring the potential of multi-agent reinforcement learning for practical automated software engineering.

**Figure 3:**
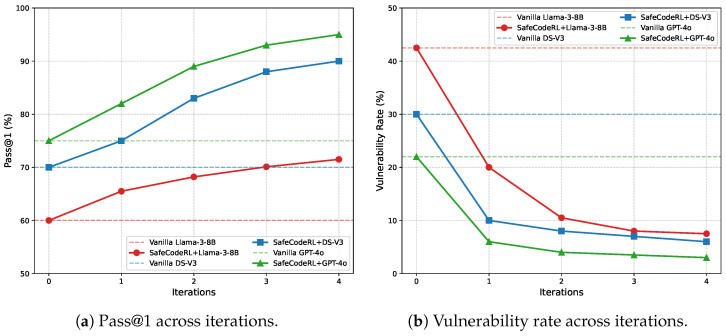
Performance and security trends of SafeCodeRL across iterations. (**a**) Pass@1 trends across refinement iterations. (**b**) Vulnerability-rate trends across refinement iterations.

As illustrated in Figure 3a, the iterative interactions among multiple agents clearly demonstrate the bidirectional optimization trajectory of code quality enabled by SafeCodeRL. Panel (a) shows that, as the number of iterations increases, the Pass@1 metric for all base models consistently rises, indicating that the Code Agent effectively assimilates functional feedback from the Test Agent and progressively repairs logical defects. Stronger base models such as GPT-4o and DeepSeek-V3 exhibit higher performance ceilings and faster convergence.

Figure 3b highlights that the vulnerability rate experiences a sharp decline within the first one to two iterations. For instance, the initial vulnerability rate of Vanilla Llama-3-8B, which starts at 42.5%, is rapidly suppressed to approximately 10% after two rounds of Critic auditing and code refinement. This convergence dynamic strongly validates that the Security Agent’s dual-audit mechanism precisely identifies vulnerability blind spots. Moreover, the dynamic reward shaping guided by the Lagrangian multiplier imposes effective penalties on high-risk actions during the early training and interaction phases, forcing the model to switch rapidly to a secure generation manifold $G_{safe}$.

Crucially, this substantial enhancement in security does not compromise functional correctness, effectively overcoming the traditional alignment tax commonly observed in code generation tasks.

### 4.4. IoT/CPS Software Security Case Study

To evaluate whether SafeCodeRL can be applied to sensor-driven software beyond generic programming benchmarks, we construct a compact IoT/CPS case-study suite with 25 code-generation tasks. The suite contains five task categories, each with five prompts: (i) sensor telemetry database/API code, where the dominant risk is SQL injection; (ii) edge-gateway diagnostic utilities, where the dominant risk is OS command injection; (iii) firmware, configuration, or log-file handlers, where the dominant risk is path traversal; (iv) secure sensor-data aggregation and authentication routines, where the dominant risk is hard-coded credentials or insecure authorization; and (v) networked sensor parsers, where the dominant risk is unsafe parsing, deserialization, or denial-of-service style boundary failures. The tasks are intentionally scenario-level rather than a full IoT benchmark; their purpose is to verify whether the general SafeCodeRL mechanism transfers to representative sensing-system software. To make the construction procedure transparent, Appendix A provides representative task examples, complexity descriptors, validation signals, and the release-status explanation for the 25-task IoT/CPS suite.

In addition to single-agent vanilla generation and Self-Refine [31], we include four representative multi-agent code-generation baselines: ChatDev [11], MetaGPT [12], MapCoder [29], and AgentCoder [13]. To control for backbone capability, these multi-agent baselines use GPT-4o [44] as the base model. They therefore test whether generic multi-agent orchestration alone is sufficient for IoT/CPS software security, as opposed to the explicit security-constrained optimization used in SafeCodeRL.

As shown in Table 3, the reported values are mean point estimates over five independent runs. SafeCodeRL provides a stable security advantage in the IoT/CPS case study: generic multi-agent methods improve functional correctness through collaborative decomposition, review, or testing, with AgentCoder reaching 91.3% Functional Pass@1 and 79.7% Secure Pass@1, but its vulnerability rate remains 15.9%. This indicates that generic multi-agent coordination does not by itself provide sufficient protection for IoT/CPS trust boundaries.

SafeCodeRL achieves stronger security because its multi-agent workflow is coupled with CWE-level auditing, CMDP-based security costs, Lagrangian penalty adaptation, and shielding. The GPT-4o configuration has the highest mean Secure Pass@1 of 89.3%, while the DeepSeek-V3 configuration reaches 84.7% Secure Pass@1 and a low vulnerability rate of 4.9%. For the compact 25-task IoT/CPS suite, the 95% confidence intervals are [87.4, 91.2] for SafeCodeRL with GPT-4o, [82.7, 86.7] for SafeCodeRL with DeepSeek-V3, and [77.6, 81.8] for AgentCoder on Secure Pass@1. Compared with AgentCoder, SafeCodeRL with GPT-4o is supported by paired task-level permutation tests for both Secure Pass@1 (p=0.006) and vulnerability rate (p=0.004). Because the suite contains only 25 tasks, we interpret the difference between the two strongest SafeCodeRL backbones cautiously; the robust conclusion is the consistent security advantage of explicit constraints over single-agent, self-refinement, and generic multi-agent baselines.

**Figure 4:**
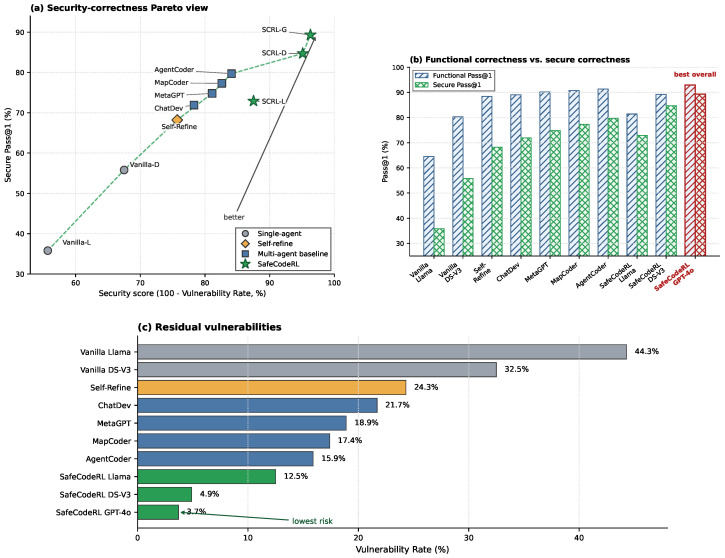
IoT/CPS case-study comparison against single-agent, self-refinement, and multi-agent code-generation baselines. (**a**) Security-correctness Pareto view. (**b**) Functional Pass@1 and Secure Pass@1 with consistent bar encodings; the red label marks the best overall configuration. (**c**) Residual vulnerability rate, where lower is better.

Figure 4 further highlights the security-oriented trade-off. In the Pareto view, SafeCodeRL configurations occupy the upper-right region, where secure correctness is high and residual vulnerability is low. The grouped-bar view shows that generic multi-agent methods narrow the gap between Functional Pass@1 and Secure Pass@1, but they still leave a visible security deficit compared with SafeCodeRL. The vulnerability-rate panel confirms that the largest reductions come from explicit security constraints rather than from agent collaboration alone. This improvement is particularly important for IoT/CPS deployment because a functionally correct but vulnerable telemetry or gateway component remains unsafe for real systems. Thus, this case study supports the claim that SafeCodeRL can serve as a trustworthy AI-assisted development layer for sensor networks, edge computing, and CPS software.

### 4.5. Efficiency and TTS Scalability

In recent years, leveraging test-time compute to boost performance has become a consensus in model deployment [48]. Test-time compute strategies can be broadly categorized into two paradigms: internal TTS exemplified by DeepSeek-R1 [45], which induces the model to generate extended reasoning chains via reinforcement learning, and external TTS represented by Best-of-N sampling with process-reward models [49].

To investigate whether multi-agent interactions under SafeCodeRL offer advantages in computational efficiency, we compare single-agent models, traditional Best-of-N sampling [49], large reasoning models such as DeepSeek-R1 [45], and SafeCodeRL on the Qwen2.5-Coder backbone [50] in terms of token consumption and performance, as shown in Table 4. DeepSeek-R1 [45] is chosen as the ultimate baseline because it represents the highest level of internal test-time compute currently achievable.

**Figure 5:**
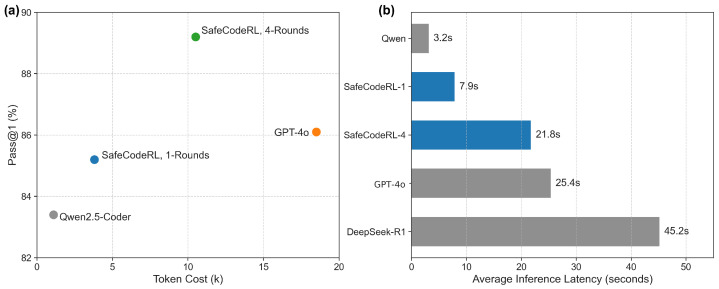
(**a**) Cost-effectiveness and (**b**) latency analysis of SafeCodeRL compared to state-of-the-art baselines.

As shown in Table 4 and the Pareto front in Figure 5a, SafeCodeRL demonstrates exceptional cost-performance efficiency. Because Table 4 mixes different scaling paradigms, we report the raw token, latency, and Pass@1 values without best/second-best highlighting. Compared to DeepSeek-R1 [45], which consumes 35,200 tokens for black-box internal trial-and-error, SafeCodeRL driven by Qwen2.5-Coder [50] achieves higher functional performance with only 10,500 tokens, reducing token consumption by approximately 70%. Furthermore, Figure 5b shows that the total latency of four multi-agent iterations is 21.8 s, which is less than half of the 45.2 s required by a single pass of the DeepSeek-R1 [45] model. These results highlight that SafeCodeRL significantly improves computational efficiency while maintaining superior Pass@1 performance. The latency numbers include Security-Agent analysis time. In the four-iteration SafeCodeRL setting, AST inspection and CodeQL-based static analysis averaged 1.3 s per Security-Agent call, or approximately 5.1 s of the 21.8 s end-to-end latency. Thus, static analysis accounts for about 23.4% of the measured runtime; at larger scale, this cost can be reduced through parallel task scheduling, AST caching, and incremental analyzer reuse, although it remains dependent on code size and rule-set complexity.

These results indicate that for code generation tasks requiring rigorous logical verification, external collaboration guided by a Critic provides higher intelligence density and time efficiency than purely internal reasoning chains. The multi-agent iterative paradigm allows SafeCodeRL to strategically allocate cognitive load across specialized agents, resulting in accelerated convergence in both functional correctness and security, while maintaining minimal computational overhead.

### 4.6. Ablation Study

To rigorously assess the contributions of individual components in SafeCodeRL, we conducted a comprehensive ablation study using the Qwen2.5-Coder-32B base model [50]. The ablation study covers architectural decomposition, training-stage choices, reward design, routing, and safety-constraint mechanisms. Table 5 summarizes the impact of removing or modifying key modules on functional performance, security robustness, and iteration efficiency.

As shown in Table 5, the full SafeCodeRL framework achieves the highest resolution rate while maintaining the lowest vulnerability rate, demonstrating the synergistic effect of its modules. Reducing the workflow to a 3-agent Code-Security-Test pipeline lowers the resolution rate to 10.9% and increases SecurityEval VR to 14.3%, showing that the Planner and Critic roles provide more than superficial workflow modularity. Removing SFT warm-start reduces the resolution rate to 10.7%, indicating that a functional coding prior is important before security-constrained reinforcement learning begins. Without Router distillation, the average number of iterations increases to 3.5, and replacing the progressive functional reward with a binary reward reduces the resolution rate to 10.4%, confirming that learned routing and milestone-level feedback stabilize the optimization process. Removing the Critic Routing mechanism forces the system into a rigid linear pipeline, resulting in a 2.6% drop in resolution rate and a near doubling of average iteration rounds. This indicates that dynamic routing effectively prioritizes the most informative agent calls, reducing redundant or ineffective interactions.

Eliminating the Lagrangian soft constraint dramatically increases the vulnerability rate to 17.5%, highlighting that the dynamic λ multiplier is critical for enforcing safety alignment without compromising functional performance. The iteration count remains low, but the functional gains are offset by a surge in unsafe actions, illustrating the necessity of adaptive reward shaping in high-risk code generation.

Removing the Shielding hard constraint causes a moderate increase in vulnerability to 11.8%, with only slight degradation in resolution. This confirms that while soft constraints guide the model’s expected behavior, physical-level safeguards are indispensable to prevent catastrophic failures during early exploration and out-of-distribution prompts.

**Figure 6:**
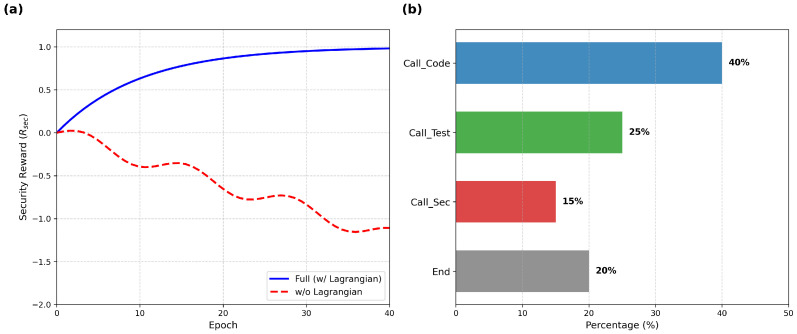
Effectiveness of dynamic constraint adjustment and action routing in SafeCodeRL. (**a**) Five-seed average convergence of the normalized security reward ($R_{sec}$) with and without the dynamic Lagrangian multiplier; negative values indicate that vulnerability costs exceed the clean-code reward. (**b**) Action distribution executed by the Critic Routing module, demonstrating a balanced allocation between code generation, testing, and security verification.

Figure 6 provides additional insights into the underlying mechanisms. Figure 6a shows that the dynamic Lagrangian multiplier λ quickly restores the security reward to positive levels during the mid-training phase, ensuring high-risk actions are penalized effectively. The y-axis is the normalized security reward $R_{\sec}$; negative values in the w/o Lagrangian curve indicate that vulnerability costs exceed the clean-code reward when the multiplier is fixed. The curve in Figure 6a reports the averaged trend over five seeds, rather than a single favorable run, and is consistent with the small final-λ variance reported above. Figure 6b illustrates the action distribution under Critic Routing, revealing that the system no longer follows a rigid Code-Security-Test sequence. Instead, high-value trajectories such as Call_Code and Call_Test dominate, while unnecessary Call_Sec and terminal calls are minimized. This optimized routing not only accelerates convergence but also enhances both functional and security outcomes.

Overall, the expanded ablation study shows that SafeCodeRL’s performance is not attributable to a single module: the five-agent decomposition, SFT warm-start, Router distillation, progressive functional reward, dynamic Lagrangian soft constraint, Critic-based routing, and hard Shielding jointly support functional correctness, security robustness, and iteration efficiency.

### 4.7. Sensitivity Analysis

To validate the robustness of the SafeCodeRL framework under different component configurations, we conducted sensitivity analyses along two key dimensions: Critic base model capabilities and the shielding mechanism’s defensive distribution.

#### 4.7.1. Critic Base Model Capability Sensitivity

In a multi-agent framework, the corrective guidance capability of the Critic largely determines the performance ceiling of the Generator. We fixed the Code Agent (Generator) as Qwen2.5-Coder-32B [50], specialized in code generation but weak in security awareness, and selected Critic models with varying parameter scales and reasoning capabilities, including Llama-3-8B [42], DeepSeek-V3 [43], and GPT-4o [44], for cross-combination experiments. Table 6 shows the functional and security performance under different Critic bases.

**Figure 7:**
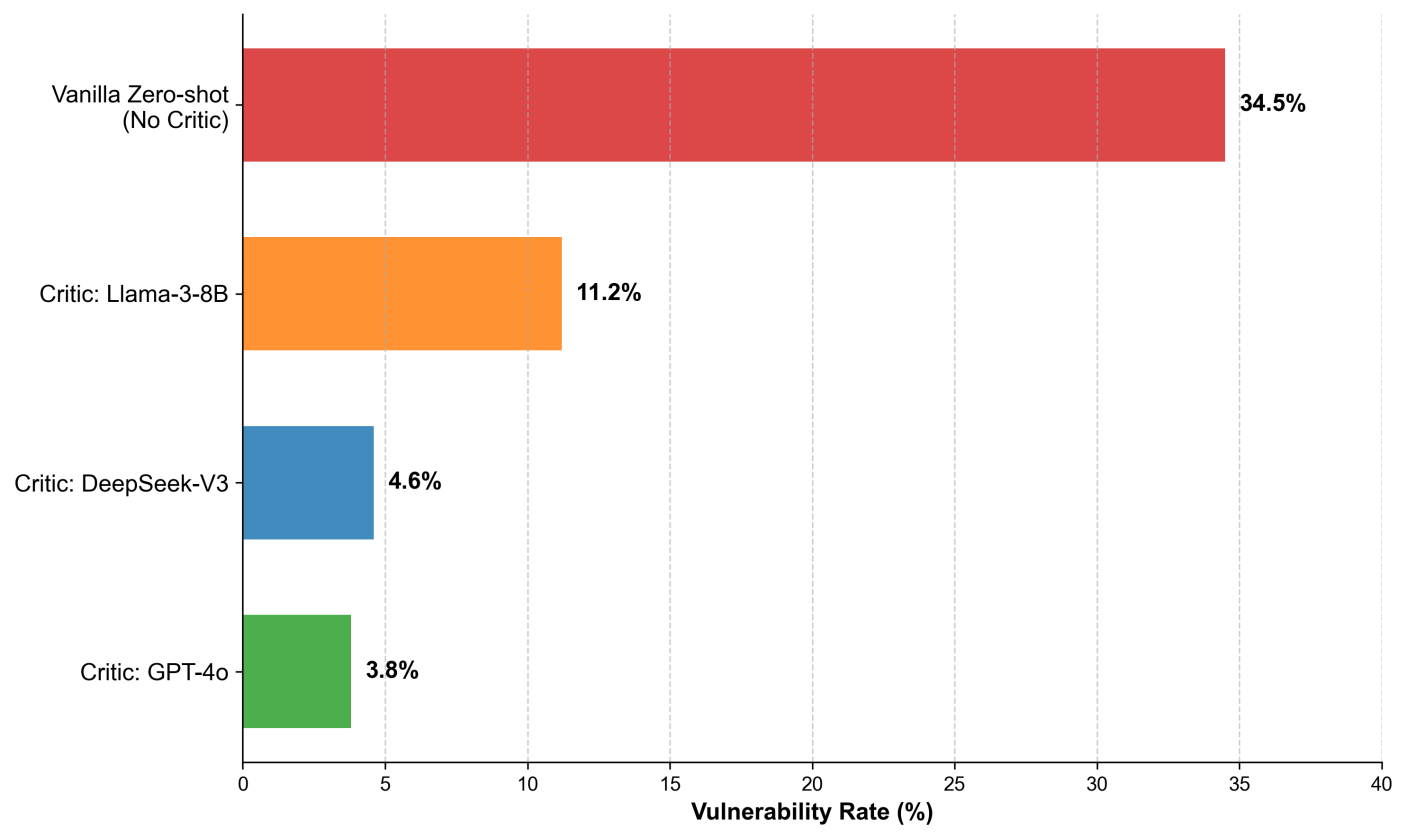
Suppression effect of different critic baselines on VR.

As illustrated in Figure 7, the choice of Critic base model exerts a decisive influence on the generator’s final vulnerability rate (VR). Without any Critic, the Vanilla Zero-shot generator exhibits a high VR of 34.5%, indicating that the generator alone is insufficient to mitigate security risks. This no-Critic value is now reported explicitly as the first row of Table 6. Introducing a weaker Critic such as Llama-3-8B [42] provides basic corrective guidance, reducing VR to 11.2%. Leveraging stronger Critic models, DeepSeek-V3 [43] and GPT-4o [44], further suppresses VR to 4.6% and 3.8%, respectively, achieving absolute reductions of over 30 percentage points compared to the Zero-shot baseline. These results highlight that a high-capability Critic can effectively elevate the security floor of even a specialized generator with limited safety awareness. Through dense interactive corrections and reinforcement learning reward signals, the framework transfers the security-aligned reasoning of the Critic to the generator, enabling rapid convergence to a safe generation manifold without compromising functional correctness. Compared to traditional single-agent generation, SafeCodeRL demonstrates a 7–9× reduction in high-risk vulnerabilities while simultaneously improving functional correctness metrics such as Pass@1 and resolution rate. This underscores the critical role of multi-agent collaboration in high-stakes code generation tasks and quantitatively confirms the substantial security and performance advantages of the SafeCodeRL paradigm.

#### 4.7.2. Shielding Trigger Distribution

**Figure 8:**
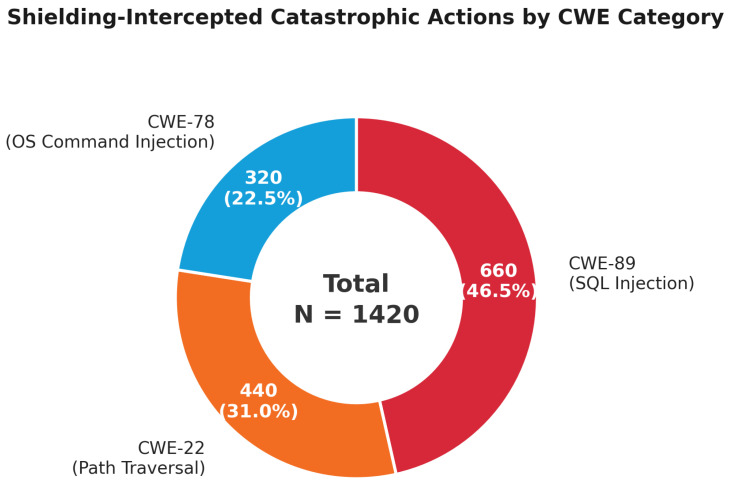
Distribution of Shield-triggered catastrophic action categories (N=1420 total interceptions). Each slice is annotated with both the absolute count and the relative percentage. This figure visualizes the operational scope of the hard Shield rather than broader CWE coverage. CWE-78: OS command injection (320, 22.5%), CWE-89: SQL injection (660, 46.5%), CWE-22: Path traversal (440, 31.0%).

While the Lagrangian soft constraint guides the PPO policy toward safety at a macro level, physical hard constraints provided by the Shielding mechanism are critical under extreme out-of-distribution prompts or highly adversarial inputs. The Shield is deliberately implemented as a narrow hard-safety layer for a small set of catastrophic actions, rather than as a general CWE detector. We therefore do not claim that a fixed Shield blacklist is a complete defense against all adversarial obfuscations; robustness comes from the layered combination of Security-Agent auditing, Critic/Lagrangian penalties, sandbox tests, and hard Shielding for selected high-impact actions. We recorded a total of N=1420 potentially catastrophic actions intercepted by Shielding in the sandbox throughout all test cycles. To make the distribution fully reproducible, we report both the absolute interception counts and their relative proportions in Table 7, and visualize them in Figure 8. Table 7 and Figure 8 therefore report the trigger distribution and operational scope of the Shield, rather than evidence of coverage over the broader CWE landscape.

As shown in Table 7 and Figure 8, SQL injection (CWE-89) is the most frequent attempted dangerous operation, with 660 interceptions (46.5% of all blocked actions); path traversal (CWE-22) follows with 440 interceptions (31.0%); and OS command injection (CWE-78) accounts for 320 interceptions (22.5%). This distribution should be interpreted as a diagnostic record of which hard Shield rules were activated, not as proof that the Shield alone generalizes to the full CWE taxonomy. Broader vulnerability mitigation in SafeCodeRL is supported by the Security Agent’s CWE-level auditing, Critic feedback, benchmark-level SecurityEval/CyberSecEval vulnerability-rate reductions, and the IoT/CPS Secure Pass@1 analysis. Shielding is therefore a complementary hard operational boundary against a small set of catastrophic actions, rather than a replacement for broader CWE-level vulnerability detection.

**Summary:** The sensitivity analysis shows that SafeCodeRL’s security improvements are not attributable to a single component. Stronger Critic models inject higher-quality CWE-level feedback into the reinforcement learning loop, whereas action-level Shielding bounds a deliberately narrow set of catastrophic behaviors such as unsafe SQL construction, path traversal, and shell execution under adversarial or out-of-distribution prompts. These two mechanisms are complementary: the Critic shapes the policy toward safer generations across broader vulnerability patterns, and Shielding preserves a hard operational boundary against selected high-impact actions.

## 5. Ethics Statement

Endowing large language models with advanced code generation and autonomous repair capabilities raises profound ethical concerns. SafeCodeRL is designed defensively to assist developers in automatically patching vulnerabilities and resisting external attacks. However, the disclosed multi-agent reinforcement learning mechanisms and vulnerability feature extraction methods could theoretically be misused by malicious actors, for example by inverting safety rewards to train a system that automatically identifies software weaknesses and generates targeted exploits. We strongly advocate for strict access control over adversarial training sandboxes and for rigorous release audits of RL-optimized code generation models, ensuring that high-intelligence automation tools such as SafeCodeRL serve only positive goals in software security.

## 6. Discussion

The results suggest that trustworthy LLM-generated software for sensor-driven systems should be treated as a constrained optimization problem rather than as a post-generation filtering task. Single-agent generation and self-refinement can improve functional correctness, but they provide weak guarantees when untrusted sensor inputs interact with high-impact resources such as telemetry databases, configuration files, edge-gateway commands, and aggregation endpoints. SafeCodeRL addresses this gap by coupling role-specialized agents with CMDP-based security costs, sandbox feedback, and action-level Shielding, so that functional progress and vulnerability suppression are optimized within the same learning loop. This design is especially relevant to IoT/CPS settings, where generated code may become part of a sensing, control, or data-aggregation path rather than a standalone programming artifact.

The current evidence should also be interpreted within its experimental scope. The IoT/CPS study is scenario-level and software-only: it uses mocked sensor streams, virtual device paths, and sandboxed gateway commands rather than physical sensors, embedded firmware, MQTT/CoAP deployments, hardware-in-the-loop testbeds, or live industrial control loops. The framework also relies on external analyzers such as CodeQL [32] and containerized sandboxes such as Docker [34], which introduce additional rollout overhead. For practical edge or embedded deployment, SafeCodeRL is best interpreted as an off-device or edge-gateway development-time/revision-time assistant: lightweight generated code can run on constrained devices, whereas Security-Agent analysis, Critic routing, and multi-round sandbox validation should be executed on an edge gateway, CI server, or workstation with caching and parallel scheduling to control latency. Moreover, hard Shielding may reject uncommon but legitimate implementation patterns in resource-constrained embedded environments. These limitations do not change the main finding, but they delimit the claims to secure code generation under sandboxed and deployment-inspired IoT/CPS software scenarios, rather than end-to-end safety certification for deployed physical systems.

## 7. Conclusions and Future Work

This paper presented SafeCodeRL, a security-constrained multi-agent reinforcement learning framework for trustworthy LLM-generated IoT/CPS software. By combining a five-agent workflow with CMDP modeling, Lagrangian security penalties, CWE-level feedback, sandbox execution, and hard Shielding, SafeCodeRL improves secure code generation without reducing the practical utility of generated programs. The broader contribution is a framework that aligns functional correctness with operational safety for sensing-system software, where vulnerabilities such as SQL injection, path traversal, command injection, unsafe authentication, and malformed-input handling can affect telemetry storage, gateway diagnostics, configuration management, and secure data aggregation. Future work will extend the evaluation from scenario-level software validation to hardware-in-the-loop sensor-network and edge-gateway testbeds, incorporate domain protocols and middleware such as MQTT, CoAP, OPC UA, and ROS, and evaluate generated code against physical sensors, embedded firmware, and realistic protocol traffic. Another promising direction is adaptive Shielding, which could learn context-aware exceptions while preserving strict boundaries against catastrophic actions.

## Figures and Tables

**Table 1:** IoT/CPS software contexts modeled in SafeCodeRL for scenario-level software validation and their corresponding validation signals.

IoT/CPS Context	Generated Artifact	Security Risks Constrained by SafeCodeRL	Functional Validation Signal
Sensor telemetry service	HTTP or database access logic for storing sensing streams	SQL injection, unsafe query construction, missing input validation	Correct insertion, retrieval, and aggregation over mocked telemetry records
Edge gateway diagnostics	Python or shell-wrapper utility for local device status checks	OS command injection, unsafe subprocess calls, unrestricted external commands	Correct parsing of diagnostic requests under sandboxed execution
Firmware/config. handler	File upload, update, and log-access logic	Path traversal, unrestricted filesystem access, hard-coded secrets	Correct file selection and update behavior within a restricted virtual device tree
Secure data aggregation	Local aggregation or filtering function for multi-sensor inputs	Insecure authentication, unsafe trust assumptions, leakage of device identifiers	Accurate aggregation under normal and boundary sensor streams
Networked sensor parser	Parser for MQTT/HTTP-style payloads or edge messages	Denial-of-service style parsing errors, unsafe deserialization, malformed-input crashes	Robust parsing of valid, missing, malformed, and adversarial payloads

**Table 2:** Quantitative comparison of different frameworks, generic multi-agent baselines, and SafeCodeRL on functional and security benchmarks. Values are mean point estimates over five independent runs. Here, ↑ indicates higher is better and ↓ indicates lower is better.

Method	Agent Base Model	HumanEval Pass@1 ↑	SWE-Bench Resolution ↑	SecurityEval VR ↓	CyberSecEval VR ↓
Single-Agent Vanilla [42]	Llama-3-8B	62.4%	3.1%	42.5%	38.7%
Single-Agent Vanilla [43]	DeepSeek-V3	75.2%	8.2%	32.1%	29.5%
Single-Agent Vanilla [44]	GPT-4o	85.1%	8.7%	29.8%	26.5%
Single-Agent Reasoning [45]	DeepSeek-R1	82.5%	12.4%	15.6%	12.8%
Self-Refine [44]	GPT-4o	88.2%	11.2%	24.3%	22.1%
ChatDev [11]	GPT-4o	88.6%	11.6%	21.9%	19.8%
MetaGPT [12]	GPT-4o	89.1%	12.0%	20.4%	18.7%
MapCoder [29]	GPT-4o	89.8%	12.7%	18.9%	17.3%
AgentCoder [13]	GPT-4o	90.2%	13.1%	17.6%	16.4%
SafeCodeRL (Ours)	Llama-3-8B	78.5% (+16.1)	8.5% (+5.4)	12.2% (−30.3)	14.8% (−23.9)
SafeCodeRL (Ours)	DeepSeek-V3	86.8% (+11.6)	15.2% (+7.0)	4.2% (−27.9)	5.8% (−23.7)
SafeCodeRL (Ours)	GPT-4o	91.4% (+6.3)	14.8% (+6.1)	5.5% (−24.3)	6.2% (−20.3)

**Table 3:** Scenario-level IoT/CPS software security results on 25 generated-code tasks. Values are mean point estimates over five independent runs; selected confidence intervals and paired significance tests are discussed in the text. Secure Pass@1 requires both functional correctness and absence of medium- or high-severity vulnerabilities. Here, ↑ indicates higher is better and ↓ indicates lower is better.

Method	Base Model	Functional Pass@1 ↑	Vulnerability Rate ↓	Secure Pass@1 ↑	Avg. Iterations ↓
Single-Agent Vanilla [42]	Llama-3-8B	64.5%	44.3%	35.8%	1.0
Single-Agent Vanilla [43]	DeepSeek-V3	80.3%	32.5%	55.8%	1.0
Self-Refine [31]	GPT-4o	88.4%	24.3%	68.2%	2.8
ChatDev [11]	GPT-4o	89.0%	21.7%	71.9%	3.7
MetaGPT [12]	GPT-4o	90.2%	18.9%	74.8%	3.5
MapCoder [29]	GPT-4o	90.7%	17.4%	77.3%	3.2
AgentCoder [13]	GPT-4o	91.3%	15.9%	79.7%	3.1
SafeCodeRL (Ours)	Llama-3-8B	81.4%	12.5%	72.9%	2.4
SafeCodeRL (Ours)	DeepSeek-V3	89.2%	4.9%	84.7%	2.1
SafeCodeRL (Ours)	GPT-4o	92.9%	3.7%	89.3%	1.9

**Table 4:** Comparison of token consumption, inference latency, and functional performance under test-time compute extensions, covering heterogeneous scaling paradigms such as no scaling, external sampling, internal reasoning, and multi-agent collaboration. Here, ↑ indicates higher is better and ↓ indicates lower is better.

Model	Scaling Paradigm	Avg. Tokens per Problem ↓	Inference Latency (s) ↓	Pass@1 (%) ↑
Qwen2.5-Coder [50] (Single)	No Scaling	1100	3.2	83.4
GPT-4o [44] (Single)	External Sampling Best-of-N (*N* = 8)	18,500	25.4	86.1
DeepSeek-R1 [45] (Single)	Internal Long-Chain Reasoning	35,200	45.2	82.5
SafeCodeRL-Qwen (1 Iteration)	External Multi-Agent Collaboration	3800	7.9	85.2
SafeCodeRL-Qwen (3 Iterations)	External Multi-Agent Collaboration	8200	16.5	88.5
SafeCodeRL-Qwen (4 Iterations)	External Multi-Agent Collaboration	10,500	21.8	89.2

**Table 5:** Expanded ablation study of architectural, training-stage, reward, routing, and safety-constraint components in SafeCodeRL (Base model: Qwen2.5-Coder). Here, ↑ indicates higher is better and ↓ indicates lower is better.

Variant Configuration	SWE-Bench Resolution Rate ↑	SecurityEval VR ↓	Average Iterations ↓
SafeCodeRL Full	14.1%	4.6%	2.3
*w/o* Five-Agent Decomposition (3-Agent Code-Security-Test)	10.9%	14.3%	4.1
*w/o* SFT Warm-start	10.7%	8.9%	3.6
*w/o* Router Distillation	12.2%	6.8%	3.5
*w/o* Progressive $R_{\mathrm{func}}$ (Binary Functional Reward)	10.4%	9.6%	3.9
*w/o* Critic Routing (Fixed Pipeline)	11.5%	5.2%	4.0
*w/o* Lagrangian Constraint (Static Weight)	12.8%	17.5%	2.5
*w/o* Shielding (Removed Hard Constraint)	13.9%	11.8%	2.4

**Table 6:** Performance sensitivity under different Critic bases with fixed Generator (Qwen2.5-Coder [50]), including the no-Critic Vanilla Zero-shot baseline. Here, ↑ indicates higher is better and ↓ indicates lower is better.

Generator	Critic	HumanEval (Pass@1) ↑	SecurityEval (VR) ↓	Critique Quality ↑
Qwen2.5-Coder	No Critic (Vanilla Zero-shot)	83.4%	34.5%	N/A
Qwen2.5-Coder	Llama-3-8B [42]	84.5%	11.2%	3.2/5.0
Qwen2.5-Coder	DeepSeek-V3 [43]	88.7%	4.6%	4.5/5.0
Qwen2.5-Coder	GPT-4o [44]	89.5%	3.8%	4.8/5.0

**Table 7:** Absolute counts and proportions of Shield-triggered catastrophic action categories across all test cycles (N=1420). This table reports the operational scope of the hard Shield rather than coverage over the broader CWE landscape.

CWE-ID	Vulnerability Category	Count	Share (%)
CWE-89	SQL injection	660	46.5
CWE-22	Path traversal	440	31.0
CWE-78	OS command injection	320	22.5
Total	—	1420	100.0

## Data Availability

The public datasets and benchmarks used in this study are available from their original repositories: HumanEvalat https://github.com/openai/human-eval (accessed on 28 March 2026) and https://huggingface.co/datasets/openai/openai_humaneval (accessed on 28 March 2026); APPS at https://github.com/hendrycks/apps (accessed on 28 March 2026) and https://huggingface.co/datasets/codeparrot/apps (accessed on 28 March 2026); SWE-bench at https://github.com/SWE-bench/SWE-bench (accessed on 28 March 2026) and https://huggingface.co/datasets/princeton-nlp/SWE-bench (accessed on 28 March 2026); SecurityEval at https://github.com/s2e-lab/SecurityEval (accessed on 28 March 2026) and https://huggingface.co/datasets/s2e-lab/SecurityEval (accessed on 28 March 2026); and CyberSecEval/Purple Llama at https://github.com/meta-llama/PurpleLlama (accessed on 28 March 2026) and https://meta-llama.github.io/PurpleLlama/CyberSecEval/ (accessed on 28 March 2026). All resources were last accessed on 28 March 2026. The complete 25-task IoT/CPS case-study suite is not publicly released because it contains vulnerability-triggering prompts, sandbox commands, and attack-style payloads with dual-use risk; Appendix A reports the construction rules, representative tasks, complexity descriptors, and validation signals needed to interpret the results.

## Appendix A. Representative IoT/CPS Case-Study Tasks

The 25-task IoT/CPS case-study suite is constructed as five categories with five prompts per category. Each task instance contains a natural-language requirement, a mocked input schema, an explicit security boundary, functional tests, and a security oracle aligned with the dominant CWE risk. Task complexity is described by the number of exposed code interfaces, input fields, boundary tests, and security rules. Table A1 lists representative examples used to define the suite. The complete suite is not publicly released because the prompts include vulnerability-triggering payloads, sandbox commands, and attack-style test inputs with dual-use risk; instead, the construction rules, representative examples, complexity descriptors, and validation signals are reported here to support interpretation and reproducibility of the evaluation design.

**Table A1:** Representative construction examples for the 25-task IoT/CPS case-study suite.

Task Category	Representative Requirement	Complexity Descriptor	Dominant Security Oracle	Validation Signal
Sensor telemetry API	Store/query mocked sensor records.	2 interfaces; 4–6 fields; 3–5 boundary tests.	CWE-89 SQL injection.	Correct records; reject tainted telemetry.
Edge-gateway diagnostics	Parse diagnostic parameters.	1 wrapper; 3–4 parameters; command-like cases.	CWE-78 command injection.	Bounded output; no shell expansion.
Configuration or firmware path handler	Normalize virtual file paths.	1 path API; nested paths; traversal payloads.	CWE-22 path traversal.	Access stays inside virtual device tree.
Secure aggregation and authentication	Aggregate readings after credential checks.	Aggregation logic; token check; 4–8 fields.	Hard-coded credential or insecure auth.	Aggregate correctly; reject invalid credentials.
Networked sensor parser	Parse malformed sensor packets.	Parser state logic; malformed packets; DoS boundaries.	Unsafe deserialization or DoS.	Bounded errors; no unsafe deserialization.

## Appendix B. Prompt Design for Agents

This appendix lists the full prompt templates used by the five agents in SafeCodeRL (Planner, Code, Security, Test, and Critic). Table A2–Table A6 show the corresponding system prompts and user-prompt templates.

**Table A2:** Planner agent prompt design.

Prompt Component	Content
System Prompt	You are an elite Software Architect. Your primary task is to break down complex user requirements into a Directed Acyclic Graph (DAG) of atomic, executable coding subtasks.
	You must strictly output your plan in JSON format like this:
	["task_id": "T1", "dependency": [], "description": "...", "in_out_types": "...", ...]
	Do not output any markdown code blocks, explanations, or conversational text outside the JSON array.
User Prompt Template	User Requirement: {user_instruction}
	Action: Decompose this requirement into logical, sequential steps. Ensure variables and functional states naturally flow between dependencies. Output the JSON array now.

**Table A3:** Code agent prompt design.

Prompt Component	Content
System Prompt	You are an expert Developer. Your goal is to write highly efficient, functionally correct, and strictly secure code based on the provided subtask.
	If you are provided with critique/feedback from previous testing or security iterations, you must carefully modify the existing code to resolve the reported errors or vulnerabilities.
	Only output the raw, executable Python code within a “‘python block. No conversational text.
User Prompt Template	Subtask Description: {task_description}
	Current Code (if refining): {current_code}
	Critic Feedback (if any): {critic_feedback}
	Action: Generate or refine the code to fulfill the subtask. Explicitly address the Critic’s feedback if provided.

**Table A4:** Security agent prompt design.

Prompt Component	Content
System Prompt	You are a strict Cybersecurity Auditor. You will be provided with a generated code snippet and a preliminary Static Analysis Report (e.g., from AST parsing or CodeQL).
	Your task is to filter out false positives from the static report, confirm real logic vulnerabilities, and output a structured vulnerability report. Think adversarial.
User Prompt Template	Code Snippet: {code_snippet}
	Static Analysis Raw Report: {sast_report}
	Action: Analyze the data flow. If a vulnerability is confirmed, output exactly in this format:
	CWE-ID: [e.g., CWE-89]
	Severity: [Low/Medium/High]
	Vulnerable Lines: [line numbers]
	Mitigation Hint: [Concrete instructions to fix it securely]

**Table A5:** Test agent prompt design.

Prompt Component	Content
System Prompt	You are an expert Software QA Engineer. Your task is to generate comprehensive edge-case unit tests (in Python unittest or pytest) for the provided code to verify functional correctness and robustness. Ensure coverage of boundary conditions and failure points.
User Prompt Template	Target Code Snippet: {current_code}
	Action: Generate 3 to 5 edge-case test functions to stress-test the above code. Output ONLY the raw Python test code within a “‘python block. Do not write test data requiring external file reads unless mocked.

**Table A6:** Critic agent prompt design.

Prompt Component	Content
System Prompt	You are a rigorous Code Reviewer and System Router. Aggregate execution logs from the Sandbox Test and the vulnerability report from the Security Auditor.
	Generate a concise, actionable Critique for the Developer. Output a routing tag (<ROUTE: Code|Test|Security|End>) to determine the next agent.
User Prompt Template	Current Source Code: {current_code}
	Test Execution Logs: {test_logs}
	Security Auditor Report: {security_report}
	Action: Summarize critical failures. Pinpoint logic flaws and enforce secure coding practices. Provide a unified [CRITIQUE] section, followed immediately by the [ROUTING] section.
